# *Brownie*, a Gene Involved in Building Complex Respiratory Devices in Insect Eggshells

**DOI:** 10.1371/journal.pone.0008353

**Published:** 2009-12-16

**Authors:** Paula Irles, Xavier Bellés, M. Dolors Piulachs

**Affiliations:** Institut de Biologia Evolutiva (UPF-CSIC), Barcelona, Spain; Ecole Normale Supérieure de Lyon, France

## Abstract

**Background:**

Insect eggshells must combine protection for the yolk and embryo with provisions for respiration and for the entry of sperm, which are ensured by aeropyles and micropyles, respectively. Insects which oviposit the eggs in an egg-case have a double problem of respiration as gas exchange then involves two barriers. An example of this situation is found in the cockroach *Blattella germanica*, where the aeropyle and the micropyle are combined in a complex structure called the sponge-like body. The sponge-like body has been well described morphologically, but nothing is known about how it is built up.

**Methodology/Principal Findings:**

In a library designed to find genes expressed during late chorion formation in *B. germanica*, we isolated the novel sequence Bg30009 (now called *Brownie*), which was outstanding due to its high copy number. In the present work, we show that *Brownie* is expressed in the follicle cells localized in the anterior pole of the oocyte in late choriogenesis. RNA interference (RNAi) of *Brownie* impaired correct formation of the sponge-like body and, as a result, the egg-case was also ill-formed and the eggs were not viable.

**Conclusions/Significance:**

Results indicate that the novel gene *Brownie* plays a pivotal role in building up the sponge-like body. *Brownie* is the first reported gene involved in the construction of complex eggshell respiratory structures.

## Introduction

The shell of an insect egg must guarantee protection for the yolk and embryo as well as provisions for respiration and for the entry of sperm, the later function being ensured by aeropyles and micropyles, respectively [1]. Insect species which oviposit the eggs in an egg-case have a double problem of respiration as gas exchange then involves two barriers. An example of this situation is found in the German cockroach, *Blattella germanica*.

**Figure 1:**
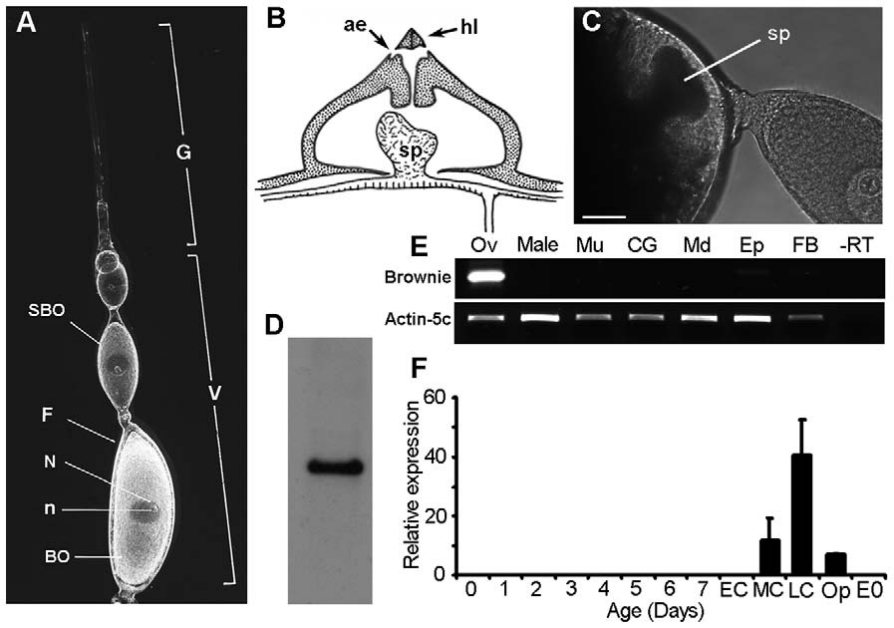
The ovariole and sponge-like body of *Blattella germanica*, and expression of *Brownie*. (A) Ovariole from the panoistic ovary of *B. germanica*; germarium (G), vitellarium (V), basal oocyte (BO), sub-basal oocyte (SBO), follicular epithelium (F), nucleus (N), nucleolus (n). (B) Scheme of the ootheca keel showing the bifurcate aeropyle (ae), the hatching line (hl) and the sponge-like body (sp) extending into the keel air cavity (modified from Hinton [1]). (C) View in toto of the anterior pole of the basal oocyte in late choriogenesis; the claviform structure is the developing sponge-like body (sp). (D) Northern Blot analysis of *Brownie* from an extract of choriogenic ovaries, showing a single band of ca. 1000 bp. (E) Tissue expression of *Brownie*; RT-PCR was carried out with RNA from adult tissues: 7-day-old ovaries during chorion formation (Ov), male whole body (male), female muscle (Mu), colleterial glands (CG), female midgut (Md), female epidermis (Ep), female fat body (FB) and ovarian tissue without reverse transcriptase as negative control (-RT); actin-5c was used as a reference gene. (F) *Brownie* expression in the ovary during the first gonadotrophic cycle; ovaries from 7-day-old adult females in the period of chorion formation were divided into three stages: EC (early choriogenesis), MC (mid choriogenesis) and LC (late choriogenesis); ovaries just after oviposition (Op) and 0-day-old embryos (E0) were also included. qRT-PCR was normalized against actin-5c. Data represent copies of mRNA per copy of actin-5c, and are expressed as the mean±SD (n = 3).

*B. germanica* is a hemimetabolan insect with panoistic ovaries that oviposits the eggs into an egg-case or ootheca. Each ovary comprises approximately twenty ovarioles formed by an array of oocytes, and within each ovariole, only the most basal oocyte matures in every gonadotrophic cycle (Figure 1A). In *B. germanica*, as occurs in insects with panoistic ovaries, each ovarian follicle is composed by one germ cell (the oocyte) surrounded by a monolayer of somatic follicular cells. As summarized by Hinton [1], the eggs of *B. germanica* have an anterior meshwork that is air-filled and projects above the surface (Figure 1B). This structure, which conveys the aeropyle and the micropyle, was well described morphologically by Wigglesworth and Beament [2] and by Lawson [3], who called it sponge-like body. During the process of chorion formation, the development of the sponge-like body is clearly visible in the late choriogenesis stage [4], [5] as a claviform structure appearing at the anterior pole of the basal oocyte (Figure 1C).

In the ootheca, the keel, along which is going the hatching line, is composed by an upper and a lower lamina making a cavity where the sponge-like body is enclosed [3] (Figure 1B). Each aeropyle in the hatching line is branched, connecting to a distinct chamber, which in turn communicates with the ambient air [1] (Figure 1B). This complex organization ensures the air exchange between the two compartments, the egg and the ootheca, and the ambient air. In this organization, the sponge-like body plays a pivotal role, but nothing is known about how it is built up. The present work reports a novel gene directly involved in the construction of such a complex eggshell respiratory structure.

## Results

### *Brownie*, a Novel Gene

With the aim of elucidating the molecular mechanisms regulating chorion formation in the cockroach *B. germanica*, we obtained an ovarian cDNA library specific for post-vitellogenesis using suppressive subtractive hybridization (SSH) [5]. We subsequently isolated a number of novel sequences from this library, including Bg30009, which was outstanding due to its high copy number.

The Bg30009 sequence resulted from assembling 91 ESTs obtained from the SSH library as well as from sequences obtained in a general ovarian cDNA library also generated in our laboratory. This assembly process retrieved a consensus sequence of 980 bp that had no significant similarity with the sequences available in databases. The consensus sequence lacked the 5′ end, and to complete it a 5′RACE was performed using the RNA Ligase Mediated Rapid Amplification of cDNA End (RLM-RACE), which is designed to amplify cDNAs only from the full-length mRNAs. We thereby obtained the complete sequence of Bg30009, which has a length of 993 bp (Accession number FN429652, Figure S1). A Northern blot analysis carried out using total RNA from choriogenic ovaries as template, and a cDNA fragment of 362 bp from the same sequence as probe, gave a specific signal with the expected size of around 1000 bp (Figure 1D). In addition, PCRs of genomic DNA gave the same sequence plus an intron of 2092 bp inserted between nucleotides 56 and 57 (Accession number FN429652, Figure S1).

The mRNA of Bg30009 has a presumed capsite at the 5′ end, and the ORF was determined by searching the Kozak sequence around the first ATG (Tables S1, S2). This approach allowed the start codon to be determined as nucleotide 47 from the 5′-end of the mRNA. The flanking nucleotides are 100% coincident with the Kozak consensus of *B. germanica* (Tables S1, S2). The program NetStart 1.0 also predicted nucleotide 47 as the translation start site.

The stop codon was localized 337 nucleotides downstream of the start codon, and the 3′UTR was found to be 611 bp long, with the polyadenylation element placed 25 nucleotides before the poly(A) tail. All these findings indicate that we obtained the full-length cDNA (Figure S1). The resulting sequence encodes for a protein of 112 amino acids with a predicted molecular mass of 12.5 kDa and an isolectric point of 5.937, and a hydrophobic character (39% of the amino acids). The protein has a neutral tandem repeat (**L**F**EE**T**PVY**G**K**Y**GIG** H **L**V**EE**I**PVY**E**K**H**GIG**), of unknown function, localized between amino acids 40 and 68, with 66% similarity between both repeats (Figure S1). As predicted by SignalP 3.0, this protein would have a peptide signal with a cleavage site between positions 21 and 22.

A new search using the complete sequence as query revealed that Bg30009 has no homologous gene or protein sequence in available databases. In light of the phenotype of the obtained knockdowns (see below), we named the new gene *Brownie*.

### *Brownie* mRNA Is Specifically Expressed in the Ovaries during Choriogenesis

The expression of *Brownie* was analyzed in a number of female tissues (ovary, muscle, midgut, epidermis, colleterial glands and fat body), as well as in male whole body extracts, by semi-quantitative RT-PCR (sqRT-PCR). The results (Figure 1E) showed that *Brownie* is specifically expressed in adult ovaries. We subsequently determined the expression of *Brownie* in the ovaries during the 7 days of the first gonadotrophic cycle by quantitative RT-PCR (qRT-PCR); on the last day, we distinguished the three previously defined stages of chorion formation [5]: early, mid and late choriogenesis (EC, MC and LC, respectively). The results (Figure 1F) indicated that *Brownie* is expressed only at the end of the cycle, showing an acute peak at the LC stage. *Brownie* mRNA is still expressed in the ovaries after the oviposition of the basal oocyte. Conversely, no expression was detected in freshly oviposited eggs, which would correspond to 0-day-old embryos, thus suggesting that *Brownie* mRNA is not delivered to the embryo maternally and is a gene expressed in the follicular epithelium.

### *Brownie* Localizes in the Follicular Cells

At MC, the follicular cells at the anterior pole of basal oocyte show a columnar aspect, leaving large intercellular spaces which increase as the formation of sponge-like body proceeds (Figure S2A). Conversely, the remaining follicular cells, in the medial part and basal pole of the oocyte, are flattened (Figure S2B). At LC the sponge-like body is finally formed in a cavity left by this particular population of columnar cells (Figure S2C).

**Figure 2:**
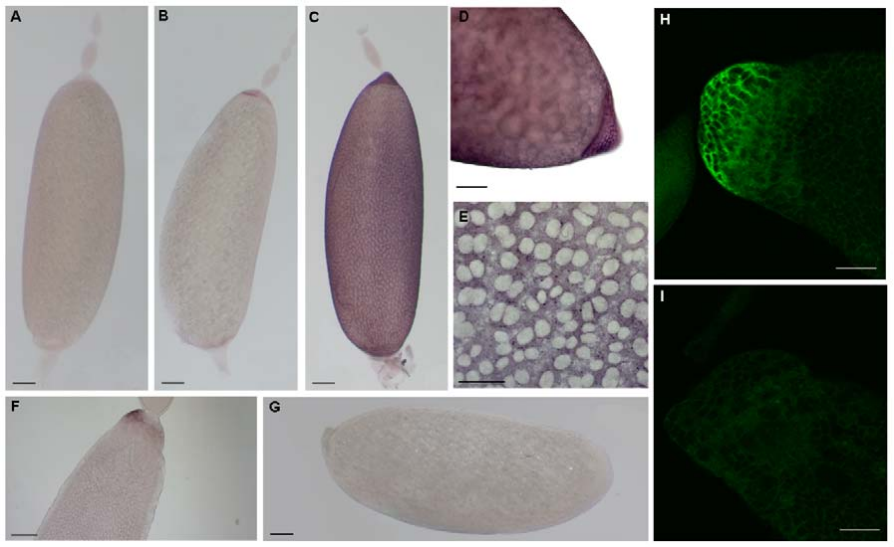
Localisation of *Brownie* mRNA in adult ovaries. In situ hybridization in whole mount ovarioles during chorion formation using sense (A) and antisense (B–G) DIG-labeled Brownie probes. (A) Negative control. (B) Mid choriogenesis stage. (C) Late choriogenesis stage. (D) Late choriogenesis stage, detail of the anterior pole. (E) Detail of cells of the anterior pole in late choriogenesis showing the label localized in the cytoplasm. (F) Epithelium sheet that remains at the basis of the ovariole after oviposition. (G) Oviposited egg. (H–I) Fluorescence in situ hybridization in the remains of the follicular epithelium after egg oviposition. Sense (I) and antisense (H) probes were labelled with Alexa Fluor® 488; the antisense probe selectively labelled (green) the cells of the anterior pole of the epithelium whereas the remaining part of the sheet, as well as the sub-basal oocyte, were not labelled (H). Negative controls using a sense probe gave no labelling (I). Scale bars: 100 µm in A–D and F–G; 20 µm in E; 25 µm in H–I.

To localize *Brownie* mRNA we carried out in situ hybridization experiments. Results indicated that there is no detectable expression at EC. Indeed, the results were identical to those observed with the sense *Brownie* probes used as negative controls (Figure 2A). *Brownie* expression began at the anterior pole of the basal oocyte at the MC stage (Figure 2B). Then, at LC, the expression clearly concentrates in the columnar cells of the anterior pole (Figure 2C, D), exactly where the sponge-like body is being developed, although a diffuse labelling is observed in the whole follicular epithelium. Examination at higher magnification revealed that expression is localized in the cytoplasm of the follicular cells (Figure 2E). *Brownie* mRNA is still detected strictly in the anterior part of the follicular sheet which remains after displacement of the egg into the oviduct (Figures 2F, H), but not in the oviposited egg (Figure 2G).

### RNAi of *Brownie* Impairs Formation of the Sponge-Like Body

**Figure 3:**
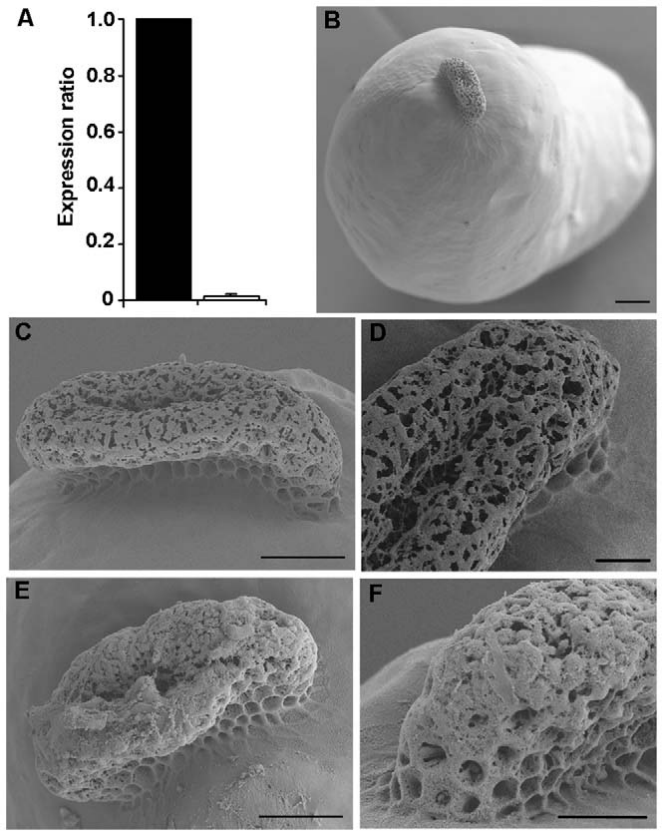
Effects of Brownie RNAi in the eggshell. (A) mRNA levels of Brownie in dsMock-treated (black column) and dsBrownie-1-treated (white column) specimens; qRT-PCR was carried out on RNA extracts from ovaries in late choriogenesis stage and in relation to the expression of actin-5c; data represent normalized values with respect to dsMock expression levels, expressed as the mean±SD (n = 3); according to the Relative Expression Software Tool 2008, *Brownie* expression in the ovaries from dsBrownie-1-treated specimens, was reduced by 67-fold with respect to dsMock group. (B) Control egg showing the sponge-like body in the anterior pole. (C–D) General view (C) and detail (D) of the sponge-like body in eggs from dsMock-treated females. (E–F) General view (E) and detail (F) of the sponge-like body in eggs from dsBrownie-1-treated females. Scale bars: 100 µm in B; 50 µm in C and E; 25 µm in D and F.

To unveil the function of *Brownie* we followed an RNAi approach. Therefore, we prepared a dsRNA encompassing a 480 bp region starting at nucleotide 128 of the *Brownie* sequence (dsBrownie-1; Figure S1), which was injected into 5-day-old adult mated females of *B. germanica* (n = 36) at a dose of 1 µg. We used a non-coding sequence from the pSTBlue-1 vector (dsMock) [6] injected at a dose of 1 µg (n = 34) as control dsRNA. The ovaries were dissected two days later, at the MC and LC stages of choriogenesis. *Brownie* mRNA levels in the ovary of dsBrownie-1-treated specimens were reduced by 67-fold with respect to dsMock-treated specimens (Figure 3A). Basal oocyte growth and colleterial glands development were not affected by the dsBrownie-1 treatment (Table 1 and Figure S3). Moreover, the typical chorion layers of the basal oocyte after chorionation were completely formed (Figure S4), only the sponge-like body showed signs of malformation. Eggs dissected from the oviducts of 10 treated and 8 control females were processed and observed by scanning electron microscopy (SEM) for a closer examination of the sponge-like body. SEM images of the controls revealed the features classically described by Wigglesworth and others [1], [2], [3], namely an elongated body, with the upper surface forming a reticulated meshwork structure with a longitudinal concavity at the top; the basis that connects with the egg shows the typical hexagonal pattern of the follicular cells (Figure 3B, D). However, most of the structure of the upper surface in the eggs from dsBrownie-1-treated specimens was covered by an amorphous accumulation of material which formed a crust resembling that of a brownie cake. Significantly, the upper longitudinal concavity, which conveys the aeropyle and micropyle conducts, was obstructed (Figure 3E, F).

**Table 1:** Oocyte growth and colleterial glands development in dsBrownie-treated females.

Treatment	BOL (mm)	LCG (µm)
dsBrownie	2.30±0.04 (n = 24)	141.80±9.90 (n = 14)
dsMock	2.29±0.03 (n = 28)	144.40±9.63 (n = 16)

Five-day-old adult females were treated with 1 µg of dsMock or dsBrownie and were dissected in late chorion stage. BOL: basal oocyte length. LCG: left colleterial gland diameter. Values are expressed as the mean±SD. Differences between treated and controls are not significant (Mann-Whitney U-statistic).

To further assess the specificity of the effects observed, we repeated these experiments using an alternative dsRNA for *Brownie*, this time targeting a 680 bp region starting at nucleotide 266, which we called dsBrownie-2 (Figure S1). A 1 µg-dose of this dsRNA was injected into 5-day-old adult females, and equivalent experiments were carried out with dsMock. The dsMock-treated specimens (n = 8) produced normal eggs whereas those treated with dsBrownie-2 (n = 12) produced eggs with a malformed sponge-like body, exactly as in the specimens treated with dsBrownie-1 (Figure S5).

### Effects on the Ootheca

**Figure 4:**
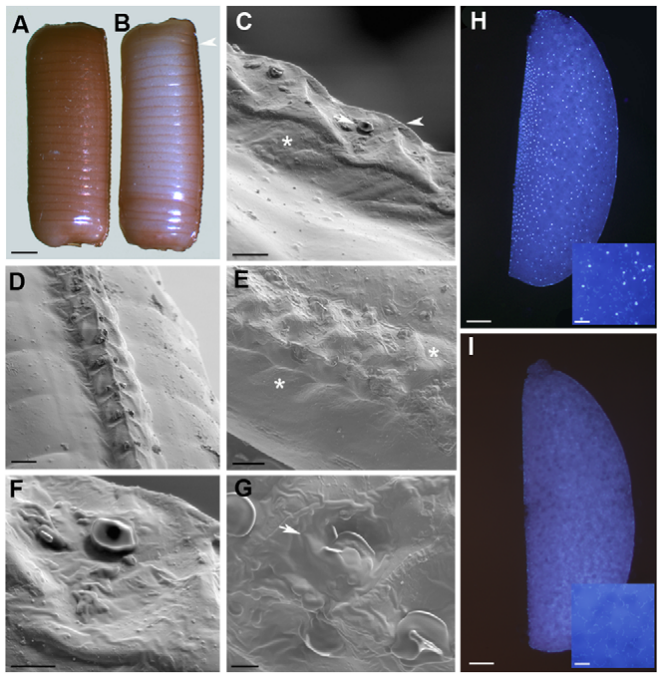
Effects of Brownie RNAi on the ootheca and eggs fate. (A–B) Lateral view of an ootheca from a dsMock-treated female (A) and from a dsBrownie-1-treated female (B), 24 h after oviposition. Complete ootheca tanning takes 5–6 h in dsMock group and 6–7 days in dsBrownie-1 group. (C) Lateral view of the ootheca keel from a ds-Mock-treated female showing the elevated keel basis (asterisk), one side aeropyle (arrow) and the hatching line at the crest of the keel (arrowhead). (D) Ootheca from a dsMock-treated female showing a uniform pattern of distribution of aeropyles in each side of the keel. (E) Ootheca from a dsBrownie-1-treated female, showing a diffuse and flattened general morphology (asterisks). (F–G) Aeropyles in oothecae from dsMock-treated specimens (F) and in dsBrownie-1-treated specimens (G), showing aeropyle obliteration in the latter group. (H) Egg from a dsMock-treated specimen 24 h after oviposition stained with DAPI; the inset shows a detail of the yolk mass with dispersed energids. (I) Egg from a dsBrownie-treated specimen 24 h after oviposition stained with DAPI; the inset shows a detail of the yolk mass without energids, showing only a faint stain due to endosymbiont bacteroids. Scale bars: 1 mm in A; 100 µm in C, D and E; 20 µm in F and G; 250 µm in H and I (and 50 µm in the insets).

To study the effects of Brownie RNAi on the ootheca, we treated a new group of mated 5-day-old females with dsBrownie-1 (n = 30) or dsMock (n = 25) and left them until the formation of the ootheca which happened 2 days later. All specimens formed an ootheca of similar size, but whereas those of dsMock-treated females reached the typical dark brown colour in 5–6 h, those produced by the dsBrownie-1-treated specimens lasted 6–7 days to reach complete tanning (Figure 4A, B). The slower tanning process in dsBrownie-1-treated specimens does not seem related to the secretion of colleterial glands, which are correctly developed (see above and Figure S3). Perhaps the malformations in the keel area and the air cavity were the sponge-like body is enclosed, along with the obliteration of the aeropyles (see below) rendered more difficult the correct oxygenation of the whole oothecal structure, thus slowering the tanning process.

For a closer examination, we selected 8 oothecae from dsBrownie-1-treated specimens and 8 from dsMock-treated specimens and studied them by SEM. Important differences were found in the keel structure, which in control oothecae appears as a prominent crest, the top of which coinciding with the hatching line, with paired and periodic aeropyles opened on both sides (Figure 4C, D, F). The ootheca from dsBrownie-1-treated specimens, on the other hand, showed the keel flattened (Figure 4E), with the periodic pattern diffused and the aeropyles distributed asymmetrically and, in most cases, obliterated (Figure 4G).

### The Fate of the Eggs in the Ootheca

The nymphs from the sample of dsMock-treated females (n = 22) hatched normally from the ootheca 17–18 days after oviposition, whereas the eggs from the dsBrownie-1-treated females (n = 22) were not viable and no nymphs emerged. The freshly oviposited egg in *B. germanica* is in metaphase stage within the first maturation division. After penetration of the sperm into the egg through the micropyle, new maturation divisions ensue and the zygote enters into a cleavage sequence. This results in cleavage energids that first spread in all directions through the yolk mass and then concentrate at the egg ventral part where, in a few hours, they form the germ band [7]. With this in mind, we carried out a DAPI staining of eggs 24 h after oviposition. In eggs from dsMock-treated females (n = 20), we observed abundant energids in the yolk mass, and already tending to concentrate in the ventral surface (Figure 4H), as expected. Conversely, the eggs from dsBrownie-1-treated females (n = 22) did not show cleavage energids in the yolk mass, and only a faint and diffuse staining due the DNA of endosymbiont bacteroids was observed (Figure 4I). Indeed, the aspect of these eggs was identical to that of virgin female eggs despite the fact that the dsBrownie-1-treated females were mated, as assessed by the presence of spermatozoids in the spermathecae.

## Discussion

*Brownie* is a novel gene with no homologues in databases. This is not surprising given that it is a gene from a cockroach with panoistic ovaries and most of the chorion-related sequences available in databases are from insects with meroistic ovaries such as dipterans and lepidopterans.

*Brownie* is expressed strictly during late choriogenesis in a particular population of follicular cells with columnar morphology located in the anterior pole of the oocyte. The sponge-like body is formed in a cavity left by these cells, when Brownie expression levels are the highest, in LC. A localized gene expression in the oocyte anterior pole, in particular in the aeropyle area, has been described in *Drosophila melanogaster* for *yellow*-*g*, *yellow-g2* [8] and *CG11381* [9], which are expressed strictly in the cells of the micropyle region at a late choriogenic stage. Parks and Spradling [10] have proposed that specific spatial expression patterns of *D. melanogaster* chorion genes may reflect gene induction on different follicular cell populations.

*Brownie* RNAi results in the formation of a defective sponge-like body. Thus, instead of the usual upper surface formed by a delicate meshwork with a longitudinal concavity, *Brownie* knockdowns show an accumulation of amorphous material. We suggest that the sponge-like body is formed by the conjunction of two or more components and further cross-linking, and that one of these components is Brownie protein. If this is the case, then the wrong proportion of components forming the sponge-like body that resulted from the RNAi of *Brownie* should have led to the observed phenotype.

The malformed sponge-like body of the *Brownie* knockdowns has important consequences on the formation of the ootheca and the fate of the eggs. The ootheca keel, for example, becomes malformed and with most of the aeropyles obliterated. The eggs were unfertilized and became non-viable, probably because the aeropyle that extends through the sponge-like body [1], [2] is obstructed.

Remarkably, the composition of the sponge-like body has been a mystery for more than fifty years. Wigglesworth [2] described the sponge-like body only by its appearance and air content, whereas Lawson [3] described it as being formed by a white substance, light in weight and very fragile. Most modern papers simply state that nothing is known about its composition [11]. The present paper therefore partially unravels this fifty-year mystery.

## Materials and Methods

### Animal Sampling

Freshly emerged adult females of *B. germanica* were obtained from a colony reared in the dark at 29±1°C and 60–70% r.h. The length of the basal oocyte was used to stage the ovaries from 0- to 7-day-old, whereas the stages of choriogenesis (EC, MC or LC) were determined according to the morphology of the anterior pole of the basal oocyte [5]. All dissections and tissue sampling were carried out on carbon dioxide-anaesthetized specimens.

### Cloning of *Brownie* cDNA

A fragment of 980 bp was previously obtained from a SSH ovarian cDNA library specific of post-vitellogenesis [Accession number: FM253364]. To complete this sequence, 5′-rapid amplifications of cDNA ends (RACE) were applied to RNA extracted from 7C ovaries using FirstChoice® RLM-RACE (Ambion, Huntingdon, Cambridgeshire, UK) according to manufacturer's instructions. The amplified fragment (primers used are detailed in Table S3) was analyzed by agarose gel electrophoresis, cloned into the pSTBlue-1 vector (Novagen, Madison, WI, USA) and sequenced.

### Amplification of the Gene Sequence

Genomic DNA was extracted from a pool of ten ovaries in the period of chorion formation (EC to LC) using DNeasy® Blood & Tissue Kit (Qiagen, Hilden, Germany), following the manufacturer instructions. Then we carried out PCR experiments to amplify the entire ORF, using the primers described in Table S3.

### RNA Extraction and Retrotranscription to cDNA

For Northern Blot analysis, total RNA was extracted from 7-day-old adult female ovaries in the period of chorion formation. For mRNA expression studies in different tissues by sqRT-PCR, total RNA was isolated from muscle, fat body, epidermis, colleterial gland and midgut tissues obtained from a pool of six adult females of different ages from 0 to 7c-day-old; RNA from a pool of male whole bodies was also included in the study. For monitoring mRNA expression by qRT-PCR, total RNA was isolated from pools of four to six ovary pairs obtained in chosen ages and stages of the first gonadotrophic cycle. All RNA extractions were performed using the Gen Elute Mammalian Total RNA kit (Sigma, Madrid, Spain). An amount of 400 ng from each RNA extraction was DNAse treated (Promega, Madison, WI, USA) and reverse transcribed with Superscript II reverse transcriptase (Invitrogen, Carlsbad CA, USA) and random hexamers (Promega). RNA quantity and quality was estimated by spectrophotometric absorption at 260 nm in a Nanodrop Spectrophotometer ND-1000® (NanoDrop Technologies, Wilmington, DE, USA).

### Northern Blot Analysis

Total RNA (10 µg) was subjected to electrophoresis in 1% agarose gels containing formaldehyde and then transferred by capillarity to a nylon membrane Whatman® (GE Healthcare, Little Chalfont, Buckinghamshire, UK) and UV cross-linked. To be used as a probe, a fragment of 362 bp was amplified by PCR and labelled with fluorescein using the Gene Images random prime-labelling module (GE Healthcare). Fluorescein labelling was detected using CDP-*Star* Chemiluminescent Substrate (Sigma), according to the manufacturer's protocol. Size of RNAs was estimated comparing with a RNA ladder (Novagen).

### Expression Studies

For monitoring the expression of *Brownie* in different tissues by sqRT-PCR, samples were subjected to PCR amplification with 40 cycles at 94°C for 30 sec, 59°C for 30 sec, and 72°C for 40 sec. Primers used are described in Table S3.

qRT-PCR was carried out to study *Brownie* expression during the first gonadotrophic cycle and to assess the effect of RNAi over mRNA levels. PCR primers used in qRT-PCR expression studies were designed using the Primer Express 2.0 software (Applied Biosystems, Foster City, CA, USA) (Table S3). The actin 5c gene of *B. germanica* (Accession number AJ862721) was used as a reference. RT-PCR reactions were performed and analyzed as previously described [5]. Statistical analysis of gene expression values was carried out using the REST 2008 program (Relative Expression Software Tool V 2.0.7; Corbett Research) [12]. This program calculates changes in gene expression between two groups, control and sample, using the corresponding distributions of *Ct* values as input. The program makes no assumptions about the distributions, evaluating the significance of the derived results by Pair-Wise Fixed Reallocation Randomization Test_tool in REST [12].

### Whole-Mount In Situ Hybridization

For in situ localisation of *Brownie* transcripts in the ovary, digoxigenin-labelled RNA probes (sense and antisense) were generated by transcription in vitro using SP6 or T7 RNA polymerases (Promega) and DIG RNA labelling mix (Roche, Barcelona, Spain), representing a 260 bp fragment. Ovarioles for whole mount procedures were dissected in PBS 0.2 M pH 6.8; fixation of the tissue and subsequent hybridization and detection reactions were carried out as previously reported [5].

For Fluorescence in situ hybridization RNA probes (sense and antisense) were 260 bp in length and were labelled with Alexa Fluor® 488, following the manufacturer's protocol (FISH Tag™ RNA Kit, Molecular Probes); the hybridization protocol for whole mount of the follicular sheet was carried out according to the method previously reported [5]; the samples were examined using the laser scanning confocal microscope (Fluoview 1000 Viever, Olympus).

### RNAi Experiments

We prepared a dsRNA encompassing a 480 bp region starting at nucleotide 128 of Brownie sequence (Figure S1) which was called dsBrownie-1. The fragment was amplified by PCR and cloned into the pSTBlue™-1. The primers used were as described above, in the section of mRNA expression studies. As control dsRNA, we obtained a 92 bp non-coding sequence from the pSTBlue-1 vector (dsMock). The preparation of the dsRNAs was carried out as described previously [6]. Five-day-old adult females were injected into the abdomen with 1 µg of dsBrownie-1 in a volume of 1 µl. Control specimens were injected with the same volume and dose of dsMock. In order to assess the specificity of the effects obtained with dsBrownie-1, we used an alternative dsRNA, dsBrownie-2, encompassing a 680 bp region starting at nucleotide 266 (Figure S1 and Table S3), which was prepared following the same methodology.

### Scanning Electron Microscopy (SEM)

Selected material, eggs and oothecae, were fixed in 2.5% glutaraldehyde in cacodylate buffer 0.2 M for at least 2 h. After rinsing twice with the same buffer, the samples were then treated with 1% osmium tetroxide at 4°C for 1 h. The tissues were dehydrated with increasing concentrations of alcohol at 15 min intervals. Finally the samples were subjected to critical-point drying in order to complete the dehydration process. The samples were attached to stubs with double-stick tape and coated with gold in a sputter-coating apparatus and observed with a Zeiss DSM940A scanning electron microscope at 15 Kvolt (Carl Zeiss MicroImaging, Barcelona, Spain).

### DAPI Staining

Eggs were dissected in Ringer's saline and fixed in 4% paraformaldehyde in PBS for 2 h. The eggs were washed with PBS 0.3% Triton (PBT) and then incubated for 5 min in 1 µg/ml DAPI in PBT. Then, they were mounted in Mowiol (Calbiochem, Madison, WI, USA) and observed using a Zeiss Axiophot microscope (Carl Zeiss MicroImaging).

## Supporting Information

Figure S1 Nucleotide and deduced amino acid sequence of Brownie of B. germanica. The mRNA has a capsite in the 5′end with the sequence TCATT (bold) that is identical to one of the four most common consensus capsites described in Drosophila melanogaster genes (Cherbas and Cherbas, 1993). The translation start site is located 47 nucleotides from the capsite, and the stop codon (asterisk) locates 337 nucleotides downstream the start site; the adenine of Kozak sequence at position −3 from the start codon is boxed; the position of the intron is signalled by an arrow; the tandem motif of 14 amino acids is underlined; the polyadenylation signal is indicated in bold and underlined.
(0.03 MB DOC)

Figure S2 Morphology of follicular cells in the anterior pole of Blattella germanica basal oocyte. (A) At MC stage the follicular cells (FC) at the anterior pole of basal oocyte show a columnar aspect, leaving large intercellular spaces. (B) The remaining follicular cells, in the medial part and basal pole of the oocyte, are flattened. (C) At LC stage the sponge-like body (sp) is finally formed in a cavity left by this particular population of columnar cells. OO: oocyte; CL: Chorion layers. Oocytes were fixed in glutaraldehide (2.5%) with cacodilate buffer (0.2M), and embebed in Spurr. Slides were stained with toluidin blue. Scale bars: 100 µm.
(0.41 MB TIF)

Figure S3 Effects of Brownie RNAi on oocyte growth and colleterial glands development. (A, B) Eggs obtained from the oviduct at oviposition time, from dsMock- (A) and dsBrownie- (B) treated females; both show the same length and degree of maturation. (C, D) colleterial glands from 6-day-old treated females treated with dsMock (C) or dsBrownie (D); both show the same degree of development. Scale bar in A and B: 0.2 mm; in C and D: 0.5 mm
(1.92 MB TIF)

Figure S4 Chorion layers of oocytes in late choriogenesis from dsBrownie-1-treated females. The chorion is composed by two basic layers, the thin inner chorion layer, which stands on the vitelline membrane, and the columnar outer chorion layer. In dsBrownie-1-treated specimens (A) the chorion layers were identical to those of dsMock group (B). The insert shows a detail of the chorion layers (1.2×). Scale bars for A and B: 2.5 µm.
(2.08 MB TIF)

Figure S5 Sponge-like body in dsBrownie-2-treated specimens. Sponge-like body of an egg from a dsMock-treated female (A), and from a dsBrownie-2-treated female (B). The phenotype of the latter is identical to that obtained with dsBrownie-1. Scale bars: 50 µm.
(1.39 MB TIF)

Table S1 Consensus of Kozak Sequence in Blattella germanica.
(0.11 MB DOC)

Table S2 Kozak consensus sequence.
(0.04 MB DOC)

Table S3 Primer pairs used for the experimental procedures
(0.04 MB DOC)
