# Supplementary material for: Brownie, a Gene Involved in Building Complex Respiratory Devices in Insect Eggshells
Source: PLoS One. 2009 Dec 16;4(12):e8353. doi: 10.1371/journal.pone.0008353 (PMC2792769; doi:10.1371/journal.pone.0008353)
Supplement: Figure S1 — Nucleotide and deduced amino acid sequence of Brownie of B. germanica. The mRNA has a capsite in the 5′end with the sequence TCATT (bold) that is identical to one of the four most common consensus capsites described in Drosophila melanogaster genes (Cherbas and Cherbas, 1993). The translation start site is located 47 nucleotides from the capsite, and the stop codon (asterisk) locates 337 nucleotides downstream the start site; the adenine of Kozak sequence at position −3 from the start codon is boxed; the position of the intron is signalled by an arrow; the tandem motif of 14 amino acids is underlined; the polyadenylation signal is indicated in bold and underlined. (0.03 MB DOC) [file pone.0008353.s001.doc]

**↓**

1 **TCATT**CAGCGAACTTTCATTCAGCATCTAACACACTTCCATCAACAATGGCAATTAAGATGATTCTAGTCGTCTC 75

1 M A I K M I L V V S 10

76 GCTGTTGGTAGCTCTGATTCAGCTCTCTGCTGCAAATGAGTGGCTTTATGGAGGATATCCTAGCATCGGTTTAGT 150

11 L L V A L I Q L S A A N E W L Y G G Y P S I G L V 35

151 AGGAGACAAGTACCTGTTTGAGGAAACTCCGGTTTACGGAAAATATGGCATTGGACACCTGGTTGAGGAAATTCC 225

36 G D K Y L F E E T P V Y G K Y G I G H L V E E I P 60

226 AGTATATGAAAAACACGGTATTGGACTCCCCTTCAGCAACACTCAGCACAAAGCCGTAGCAGTGGCCACTCCCTA 300

61 V Y E K H G I G L P F S N T Q H K A V A V A T P Y 85

301 CGAAGCTTACGCCGACGCTCAAAGCGACATCTTCAACAACTACTACAACAGCAAATATCCCGCTCACCTACCTAA 375

86 E A Y A D A Q S D I F N N Y Y N S K Y P A H L P K 110

376 AATCTACTAAAGATACAACTCATACTAACTAATAAAGAGTTGAATATCATTATTTAAAGTTTATTCTTAAATACG 450

111 I Y *

451 TACACTGACAGGGGAATAAAATCGGATACCTAATTTTTCCATTTCTCCAAATTCATAAAATTGTTATAGTCGGTG 525

526 CCAATGTTGTGTTTATTTTTGTGTATGTCATACACTGCTCAAAAAAAGTTTGGAATAATAGTCACACATGGTACT 600

601 TATCATACTGTGTTGATGAAATTGCGAATTTCACTCTATTTCAGATTATTTATTGCCCAAAATCAGAACAATGAA 675

676 CCATATTAATTTCAGTCACATTTGAGTACTGACTAAAACATATGATGGAATATACATAGTGTCATTATTCCAAAC 750

751 TTTTGTTGAGCAGTGTAGATACTAGTGACAGTGAAGATAAATTCAAATACAATCGCAATATAGCTTTATTCAAAA 825

826 TAAATTTACAATGGAAAATTTAGGTGTCCGGTTTTTATTTATTTTTTGTCTGCAGAGACATTTTGAAACATTTAT 900

901 GTACCGGTATTCTGCTCATTTTCTTTGGCATTCTTGTATTTAC**AATAAAA**CGTGAATTTTGTGACGTTAAAAAAA 975

976 AAAAAAAAAAAAAAAAAA 993
