# Supplementary figures and images for: Brownie, a Gene Involved in Building Complex Respiratory Devices in Insect Eggshells
Source: PLoS One. 2009 Dec 16;4(12):e8353. doi: 10.1371/journal.pone.0008353 (PMC2792769; doi:10.1371/journal.pone.0008353)

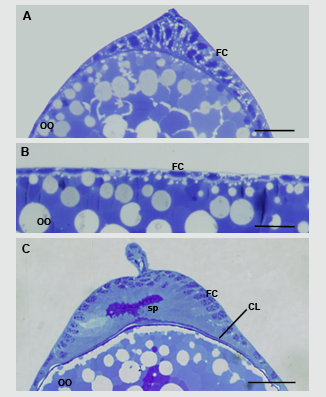

Supplement: Figure S2 — Morphology of follicular cells in the anterior pole of Blattella germanica basal oocyte. (A) At MC stage the follicular cells (FC) at the anterior pole of basal oocyte show a columnar aspect, leaving large intercellular spaces. (B) The remaining follicular cells, in the medial part and basal pole of the oocyte, are flattened. (C) At LC stage the sponge-like body (sp) is finally formed in a cavity left by this particular population of columnar cells. OO: oocyte; CL: Chorion layers. Oocytes were fixed in glutaraldehide (2.5%) with cacodilate buffer (0.2M), and embebed in Spurr. Slides were stained with toluidin blue. Scale bars: 100 µm. (0.41 MB TIF) [file pone.0008353.s002.tif]

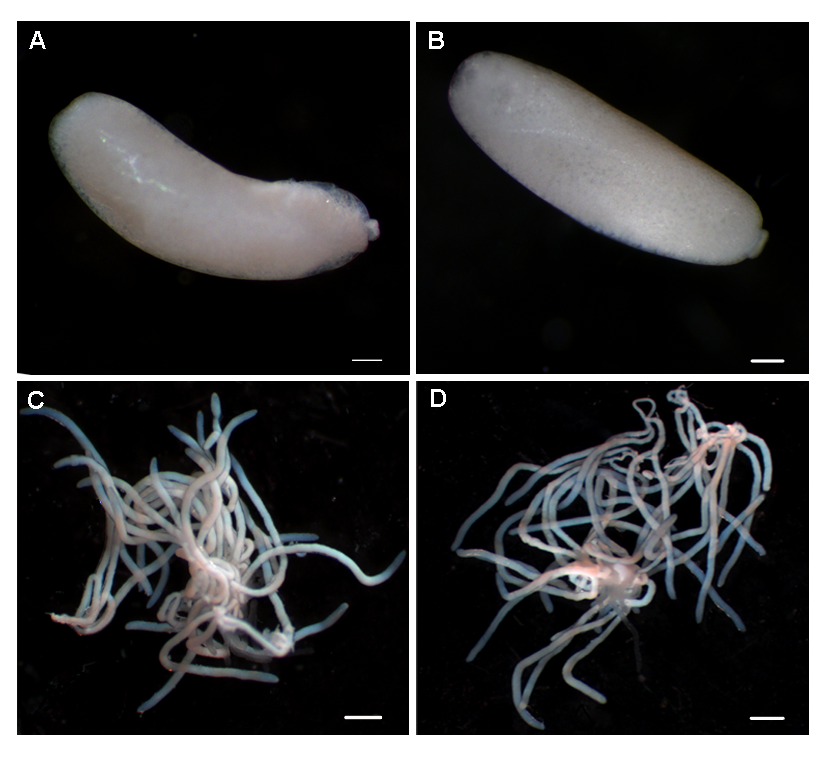

Supplement: Figure S3 — Effects of Brownie RNAi on oocyte growth and colleterial glands development. (A, B) Eggs obtained from the oviduct at oviposition time, from dsMock- (A) and dsBrownie- (B) treated females; both show the same length and degree of maturation. (C, D) colleterial glands from 6-day-old treated females treated with dsMock (C) or dsBrownie (D); both show the same degree of development. Scale bar in A and B: 0.2 mm; in C and D: 0.5 mm (1.92 MB TIF) [file pone.0008353.s003.tif]

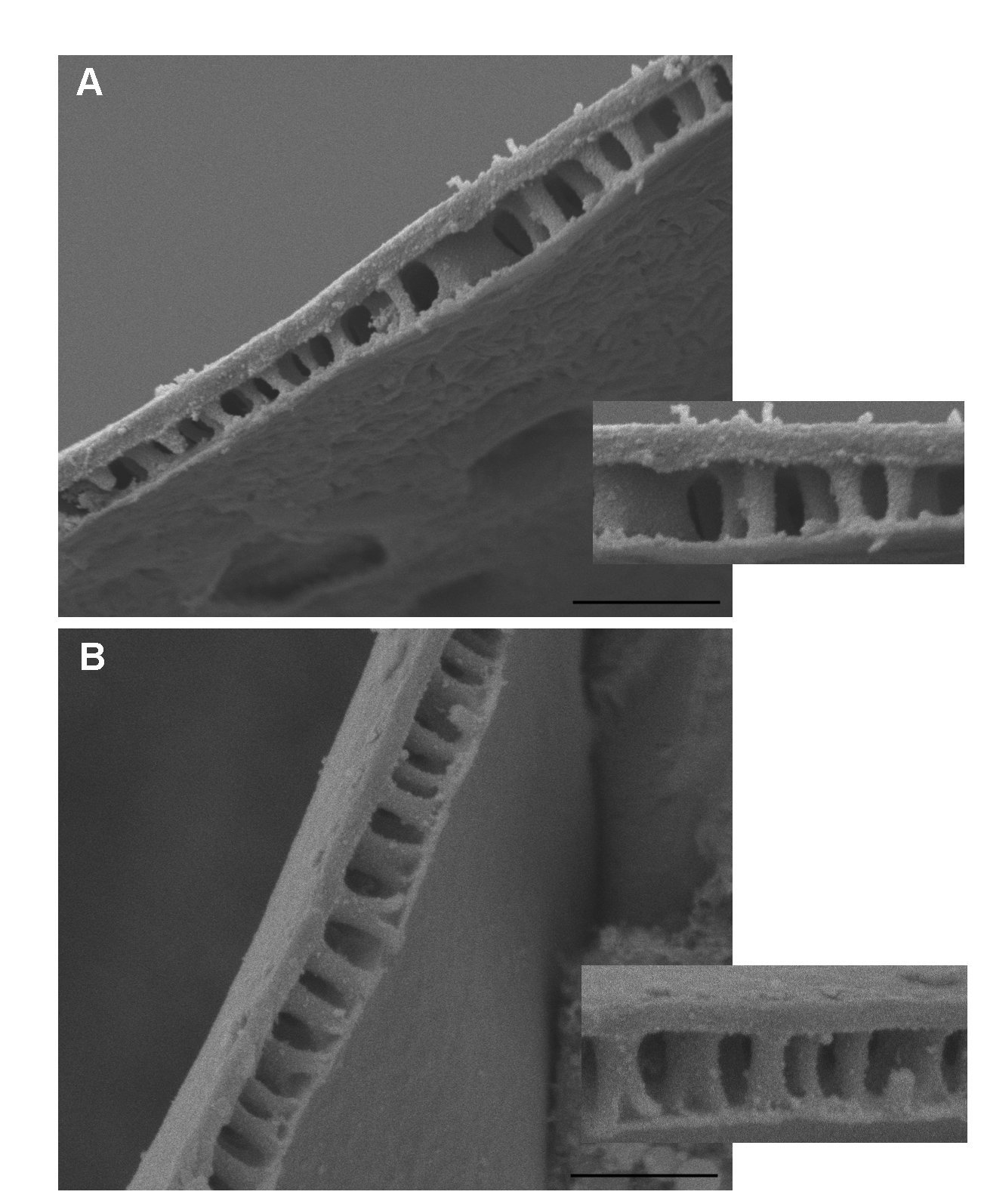

Supplement: Figure S4 — Chorion layers of oocytes in late choriogenesis from dsBrownie-1-treated females. The chorion is composed by two basic layers, the thin inner chorion layer, which stands on the vitelline membrane, and the columnar outer chorion layer. In dsBrownie-1-treated specimens (A) the chorion layers were identical to those of dsMock group (B). The insert shows a detail of the chorion layers (1.2×). Scale bars for A and B: 2.5 µm. (2.08 MB TIF) [file pone.0008353.s004.tif]

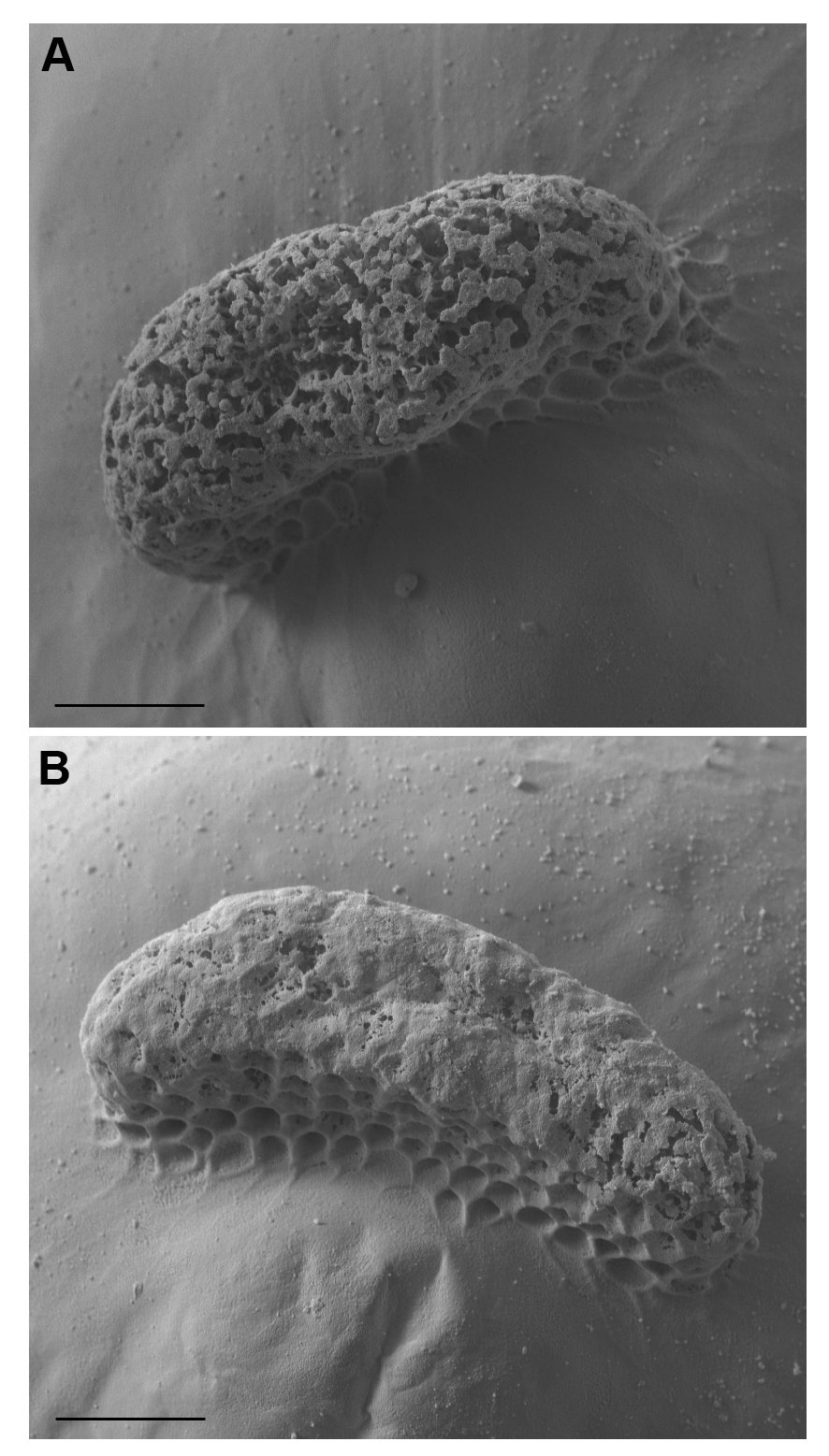

Supplement: Figure S5 — Sponge-like body in dsBrownie-2-treated specimens. Sponge-like body of an egg from a dsMock-treated female (A), and from a dsBrownie-2-treated female (B). The phenotype of the latter is identical to that obtained with dsBrownie-1. Scale bars: 50 µm. (1.39 MB TIF) [file pone.0008353.s005.tif]
