# Supplementary material for: Brownie, a Gene Involved in Building Complex Respiratory Devices in Insect Eggshells
Source: PLoS One. 2009 Dec 16;4(12):e8353. doi: 10.1371/journal.pone.0008353 (PMC2792769; doi:10.1371/journal.pone.0008353)
Supplement: Table S1 — Consensus of Kozak Sequence in Blattella germanica. (0.11 MB DOC) [file pone.0008353.s006.doc]

**Table S1. Consensus of Kozak Sequence in *Blattella germanica*.** Analysis of the flanking region of the start site of the 32 full cDNA sequences of *B. germanica* available in GenBank, in order to obtain a consensus of Kozak sequence in this species following the criteria of Cavener (Cavener, 1987). The +1 represent the first nucleotide of the start codon.

| **Gene Product** | **Accession number** | **-6** | **-5** | **-4** | **-3** | **-2** | **-1** | **+1** | **+2** | **+3** | **+4** |
| --- | --- | --- | --- | --- | --- | --- | --- | --- | --- | --- | --- |
| Cytochrome P450 CYP6K1 | [AF281328.1](http://www.ncbi.nlm.nih.gov/nuccore/14268817) | T | G | A | A | C | C | **A** | **T** | **G** | G |
| Ecdysone inducible protein 75 isoform A (E75 A) | [AM238653.1](http://www.ncbi.nlm.nih.gov/nuccore/91680595) | T | G | C | G | A | C | **A** | **T** | **G** | A |
| Pigment dispersing factor | [EU182248.1](http://www.ncbi.nlm.nih.gov/nuccore/162424762) | A | C | T | A | C | A | **A** | **T** | **G** | A |
| Mariner-like transposase | [AF355143.1](http://www.ncbi.nlm.nih.gov/nuccore/13811430) | G | T | C | A | A | C | **A** | **T** | **G** | T |
| Vitellogenin | [AJ005115.2](http://www.ncbi.nlm.nih.gov/nuccore/7009576) | T | C | C | A | A | C | **A** | **T** | **G** | A |
| Ace2 type acetylcholinesterase | [DQ288847.1](http://www.ncbi.nlm.nih.gov/nuccore/82754298) | G | G | A | A | C | C | **A** | **T** | **G** | G |
| Ace1 type acetylcholinesterase | [DQ288249.1](http://www.ncbi.nlm.nih.gov/nuccore/82754296) | G | A | A | A | A | C | **A** | **T** | **G** | G |
| Nuclear receptor (Ftz-F1 gene) | [FM163377.1](http://www.ncbi.nlm.nih.gov/nuccore/194326122) | C | A | A | A | A | C | **A** | **T** | **G** | C |
| Glutathione S-transferase | [AM778448.1](http://www.ncbi.nlm.nih.gov/nuccore/154936818) | C | T | C | A | A | G | **A** | **T** | **G** | A |
| HR3 isoform B1 | [AM259129.1](http://www.ncbi.nlm.nih.gov/nuccore/114049536) | T | G | C | G | T | C | **A** | **T** | **G** | G |
| HR3 isoform A | [AM259128.1](http://www.ncbi.nlm.nih.gov/nuccore/114049534) | T | T | G | A | C | C | **A** | **T** | **G** | T |
| Receptor for activated protein kinase C-like | [DQ885470.1](http://www.ncbi.nlm.nih.gov/nuccore/114319092) | T | A | A | A | T | T | **A** | **T** | **G** | T |
| Triosephosphate isomerase | [DQ885469.1](http://www.ncbi.nlm.nih.gov/nuccore/114319090) | G | C | A | G | A | G | **A** | **T** | **G** | G |
| Nuclear receptor (EcR-A gene) | [AM039690.1](http://www.ncbi.nlm.nih.gov/nuccore/86439689) | G | G | G | G | T | C | **A** | **T** | **G** | G |
| 1,4-α -D-glucan glucanohydrolase | [AY945930.1](http://www.ncbi.nlm.nih.gov/nuccore/62955865) | A | G | T | G | C | A | **A** | **T** | **G** | G |
| Allergen Bla g 6.0301 | [DQ279094.1](http://www.ncbi.nlm.nih.gov/nuccore/82704035) | G | A | T | A | A | A | **A** | **T** | **G** | G |
| Enolase | [DQ368397.1](http://www.ncbi.nlm.nih.gov/nuccore/86450229) | A | A | C | A | A | C | **A** | **T** | **G** | C |
| α-Amylase | [DQ355516.1](http://www.ncbi.nlm.nih.gov/nuccore/85002762) | T | C | C | G | A | G | **A** | **T** | **G** | A |
| Peptidyl-propyl cis-trans-isomerase | [X87418.1](http://www.ncbi.nlm.nih.gov/nuccore/1235942) | T | C | A | A | T | A | **A** | **T** | **G** | G |
| HMG-CoA reductase | [X70034.1](http://www.ncbi.nlm.nih.gov/nuccore/296417) | A | T | C | A | T | A | **A** | **T** | **G** | G |
| Leucomyosuppressin precursor | [AJ619986.1](http://www.ncbi.nlm.nih.gov/nuccore/46019614) | A | T | C | A | G | A | **A** | **T** | **G** | A |
| Ribosomal protein L36e | [FM253354.1](http://www.ncbi.nlm.nih.gov/nuccore/237761915) | G | T | T | A | G | G | **A** | **T** | **G** | G |
| Ribosomal protein L18A | [FM253353.1](http://www.ncbi.nlm.nih.gov/nuccore/237761913) | A | G | C | A | G | C | **A** | **T** | **G** | A |
| Yellow-g | [FM210754.1](http://www.ncbi.nlm.nih.gov/nuccore/237761897) | A | A | C | A | G | G | **A** | **T** | **G** | C |
| Target of rapamycin | [EU926975.2](http://www.ncbi.nlm.nih.gov/nuccore/206731404) | A | C | C | A | A | A | **A** | **T** | **G** | C |
| Lipophorin receptor | [AM403063.1](http://www.ncbi.nlm.nih.gov/nuccore/126540378) | A | A | G | A | C | G | **A** | **T** | **G** | T |
| Circadian clock protein (Period) | [AF297552.2](http://www.ncbi.nlm.nih.gov/nuccore/117935067) | T | T | C | A | A | G | **A** | **T** | **G** | G |
| Vitellogenin receptor | [AM050637.1](http://www.ncbi.nlm.nih.gov/nuccore/82524129) | T | C | A | A | G | A | **A** | **T** | **G** | G |
| Allatostatin neuropeptide precursor | [AF068061.1](http://www.ncbi.nlm.nih.gov/nuccore/3859876) | A | A | A | C | C | A | **A** | **T** | **G** | C |
| Retinoid X receptor | [AJ854490.1](http://www.ncbi.nlm.nih.gov/nuccore/70907500) | C | C | C | A | C | A | **A** | **T** | **G** | G |
| Dicer-1 | [FN298876.1](http://www.ncbi.nlm.nih.gov/nuccore/240247226) | C | C | T | C | T | A | **A** | **T** | **G** | G |
| HMG-CoA synthase | [X73679.1](http://www.ncbi.nlm.nih.gov/nuccore/416168) | A | A | G | A | C | G | **A** | **T** | **G** | T |
| **Consensus Kozak sequence** | | **AT** | **N** | **CA** | **A** | **N** | **AC** | **A** | **T** | **G** | **GA** |
