# Supplementary material for: Brownie, a Gene Involved in Building Complex Respiratory Devices in Insect Eggshells
Source: PLoS One. 2009 Dec 16;4(12):e8353. doi: 10.1371/journal.pone.0008353 (PMC2792769; doi:10.1371/journal.pone.0008353)
Supplement: Table S2 — Kozak consensus sequence. (0.04 MB DOC) [file pone.0008353.s007.doc]

**Table S2. Kozak consensus sequence**

Kozak sequence from the subphylum Vertebrata, the phylum Arthropoda and two insect species: *D. melanogaster* and *B. germanica*. The Kozak sequence of *Brownie* is 100% coincident with the general consensus in *B. germanica* and *D. melanogaster.*

| **Organism** | **-6** | **-5** | **-4** | **-3** | **-2** | **-1** | **+1** | **+2** | **+3** | **+4** |
| --- | --- | --- | --- | --- | --- | --- | --- | --- | --- | --- |
| Vertebrata^a^ |  |  | C | A | N | C | A | U | G |  |
| Arthropoda^b^ |  |  |  | Pu^c^ | A | A | A | U | G |  |
| *D. melanogaster*^d^ |  |  | C/A | A | A | A | A | U | G |  |
| *B. germanica*^e^ | A/T | N | C/A | A | N | A/C | A | U | G | G/A |
| *Brownie* | T | C | A | A | C | A | A | U | G | G |

^a^ Consensus obtained from Kozak (Kozak, 1984)

^b^ Consensus obtained from Kochetov (Kochetov, 2005)

^c^ Purine

^d^ Consensus obtained from Cavener (Cavener, 1987)

^e^ Consensus obtained from Table S2
