# Supplementary material for: Brownie, a Gene Involved in Building Complex Respiratory Devices in Insect Eggshells
Source: PLoS One. 2009 Dec 16;4(12):e8353. doi: 10.1371/journal.pone.0008353 (PMC2792769; doi:10.1371/journal.pone.0008353)
Supplement: Table S3 — Primer pairs used for the experimental procedures (0.04 MB DOC) [file pone.0008353.s008.doc]

**Table S3**. Primer pairs used for the experimental procedures

| **Primer name** | **Amplicon length** | **Primer sequence** | |
| --- | --- | --- | --- |
| BgActin-5c  qRT-PCR | 213 bp | F1 | 5’ AGCTTCCTGATGGTCAGGTGA 3’ |
|  |  | R1 | 5’ TGTCGGCAATTCCAGGGTACATGGT 3’ |
| BgActin-5c sqRT-PCR | 308 bp | F2 | 5’ TCGTTCGTGACATCAAGGAGAAGCT 3’ |
|  |  | R1 | 5’ TGTCGGCAATTCCAGGGTACATGGT 3’ |
| Brownie-5’Race | - | R1 | 5’ TGTAGTAGTTGTTGAAGATGTCG 3’ |
|  |  | R2 | 5’ TTGTGCTGAGTGTTGCTGAAGG 3’ |
| Brownie- Northern probe | 362 bp | F1 | 5’ AGTCACACATGGTACTTATCATAC 3’ |
|  |  | R3 | 5’ ATTGTAAATACAAGAATGCCAAAG 3’ |
| Brownie-sqRT-PCR  and dsBrownie-1 | 480 bp | F2 | 5’ GATATCCTAGCATCGGTTTAGTAG 3′ |
|  |  | R4 | 5’ GTATGATAAGTACCATGTGTGACT 3′ |
| Brownie-qRT-PCR | 51 bp | F3 | 5’ CTCAGCACAAAGCCGTAGCA 3′ |
|  |  | R5 | 5’ CGTCGGCGTAAGCTTCGTAG 3′ |
| dsBrownie-2 | 680 bp | F3 | 5’ CTCAGCACAAAGCCGTAGCA 3′ |
|  |  | R3 | 5’ ATTGTAAATACAAGAATGCCAAAG 3’ |
| Brownie-DNA | 3026 bp | F4 | 5’ CTTTCATTCAGCATCTAACA 3’ |
|  |  | R3 | 5’ ATTGTAAATACAAGAATGCCAAAG 3’ |
